# Safety of Intranasal Insulin in Type 2 Diabetes on Systemic Insulin: A Double-Blinded Placebo-Controlled Sub-Study of Memaid Trial

**Published:** 2022-06-29

**Authors:** L Aponte Becerra, B Galindo Mendez, F Khan, V Lioutas, P Novak, CS Mantzoros, LH Ngo, V Novak

**Affiliations:** 1 Department of Neurology, SAFE Laboratory, Beth Israel Deaconess Medical Center, Harvard Medical School, USA; 2 Department of Internal Medicine, Jackson Memorial Hospital, University of Miami, Miami, FL, USA; 3 Department of Neurology, Brigham and Women’s Faulkner Hospital, Harvard Medical School, Boston, MA, USA; 4 Department of Medicine, Beth Israel Deaconess Medical Center, Harvard Medical School, Boston MA and Department of Medicine, Boston VA Healthcare System, Harvard Medical School, Boston, MA, USA; 5 Department of Medicine, Beth Israel Deaconess Medical Center and School of Public Health, Harvard Medical School, Boston, MA, USA

**Keywords:** Type 2 Diabetes, Insulin, Intranasal Insulin, Safety, Hypoglycemia

## Abstract

**Aims:**

To determine safety of intranasal insulin (INI) in MemAID trial participants with diabetes treated with systemic insulins.

**Materials and Methods:**

This randomized, double-blinded trial consisted of 24-week INI or placebo treatment once daily and 24-week follow-up. Safety outcomes were: 1) Short-term effects on glycemic variability, hypoglycemic episodes on continuous glucose monitoring (CGM) at baseline and on-treatment. 2) Long-term effects on glucose metabolism and weight on INI/placebo treatment and post-treatment follow-up. Of 86 screened subjects, 14 were randomized, 9 (5 INI, 4 Placebo) completed CGM at baseline and on-treatment, and 5 (2 INI, 3 Placebo) completed treatment and follow-up.

**Results:**

INI was safe and was not associated with serious adverse events, hypoglycemic episodes or weight gain. INI administration did not acutely affect capillary glucose. Glycemic variability on CGM decreased with INI, compared to baseline. On INI treatment, there was a long-term trend toward lower HbA1c, plasma glucose and insulin. No interactions with subcutaneous insulins were observed.

**Conclusions:**

INI is safe in older people with diabetes treated with systemic insulins, and it is not associated with adverse events, hypoglycemia or weight gain. Future studies are needed to determine whether INI administration can reduce glycemic variability, improve insulin sensitivity and thus potentially lessen diabetes burden in this population

## Introduction

Type 2 diabetes mellitus (T2DM) increases risk for dementia [1]. Intranasal insulin (INI) therapy has emerged as a novel approach to treat cognitive decline. Several clinical trials have evaluated the effects of INI on cognition and functionality in T2DM [2,3], cognitive impairment and Alzheimer disease [4–6]. Previous studies have shown that acute INI administration improved verbal learning and memory and increased resting blood flow in the insular cortex in older T2DM and control participants [2,3]. Also, that INI is not associated with major side effects, including hypoglycemia (HG), both acutely and chronically [6–8]. The information regarding INI safety in individuals with T2DM treated with subcutaneous insulins is lacking.

We conducted the safety sub-study of the Memory Advancement by Intranasal Insulin in Type 2 Diabetes (MemAID) studying INI therapy in participants with T2DM on systemic insulin. MemAID was a randomized, double-blinded, placebo-controlled clinical trial, which evaluated the long-term effects of INI or placebo on cognition and gait in older T2DM and control participants. It showed that INI-treated T2DM participants walked faster, and controls had better executive functioning on INI treatment and post-treatment. Overall INI effect in the whole cohort demonstrated faster walking, and better executive function and verbal memory (p=0.02) (hemoglobin A1c-adjusted), as compared to placebo. INI treatment was not associated with serious adverse events, hypoglycemic episodes, or weight gain [9]. The INI effects in participants with T2DM on systemic insulin and potential interactions with systemic insulins are not known. We aimed to determine short-term and long-term INI safety in terms of glycemic variability, hypoglycemic episodes, and glucose metabolism as compared to placebo (sterile saline). We hypothesized that INI does not alter glycemic variability nor causes HG in T2DM participants on systemic insulin and does not affect glucose metabolism, blood pressure, or body weight.

## Materials and Methods

### Design

MemAID was a prospective double-blinded, placebo-controlled, parallel design study evaluating the long-term effects of 40 IU of human INI (Novolin® R Novo Nordisk Inc., Baksvaerd, Denmark; off label use)or sterile saline once daily on cognition and functionality in 120 T2DM, and 90 non-diabetic adults [10]. Participants were randomized into the four treatment arms (T2DM-INI, T2DM-Placebo, Control-INI, and Control-Placebo). The trial was conducted from October 6, 2015 to May 31, 2020. It was approved by the US Food and Drug Administration (FDA; IND#107690) and registered on www.clinicaltrials.gov (NCT02415556) [9,10]. This safety sub-study aimed to enroll 20 T2DM participants on systemic insulin to evaluate short-term and long-term INI safety, and the effects on glucose metabolism, vital signs, and weight. This sub-study was stopped by the Data and Safety Monitoring Board for futility due to a high drop-out rate (September 25, 2017). High drop-out rate was mostly secondary to lack of interest from participants and difficulty with adherence to the study protocol. The study was conducted at the Syncope and Falls in the Elderly (SAFE) Laboratory, Clinical Research Center (CRC) at Beth Israel Deaconess Medical Center (BIDMC), and at the Center for Clinical Investigation at Brigham and Women’s Hospital (BWH). The protocol was approved by the Committee on Clinical Investigation at BIDMC and BWH, and all participants signed the informed consent.

### Participants

Participants aged 50 to 85 years who were able to walk for six minutes were screened. Participants with T2DM on systemic insulin were required to have T2DM diagnosis and be treated with subcutaneous insulin, with or without oral or other injectable antidiabetic agents. Exclusion criteria were: type 1 diabetes, history of severe HG, serious medical conditions, hospitalization or surgery within the past six months, liver or renal failure or transplant, dementia or Mini-Mental State Examination (MMSE) score ≤20, current recreational drug or alcohol abuse. Volunteers with T2DM on systemic insulin with a C-peptide of <0.8 nmol/L and blood glucose >150 mg/dl or those with one or more episodes of HG (glucose < 70 mg/dL)11 were excluded.

### Intervention and Randomization

Participants were treated with 40 IU (0.4mL) of human insulin (rDNA origin); Novolin® R, Novo Nordisk Inc., Bagsværd, Denmark) or placebo (0.4 mL bacteriostatic Sodium Chloride 0.9% solution). Novolin® R was used off-label (https://www.novonordiskmedical.com/our-products/novolin-r.html) and was refrigerated between administrations. INI or Placebo was administered once daily before breakfast using the ViaNase device (Kurve Technologies Inc., Lynnwood WA). Devices were calibrated to dispense 0.1 ml over 20 seconds in a single dose that was administered twice into each nostril, alternating sides, over two minutes. BIDMC and BWH research pharmacy performed sterility procedures, reconstituted study drug into identical vials and dispensed according to the randomization code, without involvement in study procedures. Eligible participants with T2DM on systemic insulin were enrolled and randomized into INI and Placebo groups. The study statistician (L.N.) designed the code that used randomly selected blocks with sizes four, eight and twelve to ensure uniform distribution of baseline characteristics. The principal investigator (V.N.), co-investigators, staff, participants and participants providers were blinded to assignments. The study staff conducted the study procedures and the principal investigator and study physicians reviewed eligibility, adverse events, outcomes, and approved enrollment.

### Protocol

MemAID protocol consisted of a phone screen, screening visit (V1), baseline, four assessment visits and three follow-up visits during 24-weeks on-treatment (V2,V4,V6,V8; eight weeks apart), and four post-treatment assessment visits (V9-V12) during 24-weeks of follow-up. Assessment visits included fasting blood and glucose panels, vital signs, neuropsychological and gait testing, and adverse events evaluation. Follow-up visits included study medication refill (V3, V5, V7), device maintenance, and adverse events evaluation [10,11].

### Short-term INI Safety

Short-term INI effects on glycemic variability and hypoglycemic episodes were evaluated over five days of continuous glucose monitoring (CGM; Medtronic iPro2; Medtronic, Northridge CA, USA)) and by self-monitored blood glucose (SMBG) by five daily finger sticks before meals (Accu-Check®, Aviva PluF. Hoffman - LaRoche Ltd, Basel, CH) [12] at baseline and after INI/Placebo treatment initiation. Participants underwent SMBG education and training during baseline visit and before study intervention. Baseline CGM was inserted during on-site screening visit (V1) and the intervention CGM started at the end of first intervention visit (V2). CGM Medtronic iPro 2 is a professional CGM system with data blinded to the patient. It uses a subcutaneous small filament sensor connected to a recorder, which collects interstitial fluid to continuously measure interstitial glucose levels at five-minute intervals [13]. Short-term outcomes were interstitial and capillary glucose and glycemic variability measures. In clinical practice, professional CGM use has been shown to assist patients achieving glycemic control and modifying their life-style [14,15] So, that the 2021 American Diabetes Association (ADA) standards of care include CGM use as a useful tool to improve hemoglobin A1c (HbA1c) levels in patients with diabetes on systemic insulin regimens [16].

#### Glycemic variability (GV):

Intra-day GV was assessed by the mean amplitude of glucose excursions (MAGE), the standard deviation (SD) around the mean CGM glucose values, and continuous overlapping net glycemic action (CONGA). SD is considered the “gold standard” CGM metric and takes into account all fluctuations during CGM recording equally, whereas MAGE accounts only for major intraday oscillations [17] by adding the differences from the peaks to nadirs divided by the total number of glucose values, the difference is only considered when greater than one SD of the mean in a 24-hour period [17]. Inter-day GV was assessed by the mean of daily differences (MODD) [18]. CGM indices were calculated using the Easy GV calculator version 9.0 [19].

### Hypoglycemia Monitoring

ADA clinical practice guidelines from 2013 were used for identification of HG (symptomatic or asymptomatic hypoglycemia documented on any glucose measurement method ≤ 70 mg/dL) [11]. We excluded participants who had more than one asymptomatic and/or symptomatic episode of glucose ≤ 70 mg/dL documented on plasma glucose, SMBG or CGM during the entire study. Revised HG definition from ADA practice guidelines from 2018 and the International Hypoglycemia Study Group were adopted for data analyses and manuscript preparation as follows: 1. Level-one HG, any recorded glucose values (plasma glucose, SMBG, CGM) between ≤ 70 mg/dL and ≥54 mg/dL; 2. Level-two HG or clinically significant HG, any recorded glucose value on plasma glucose or SMBG < 54 mg/dL and, CGM glucose < 54 mg/dL for at least 20 minutes [12,20].

### Adverse Event monitoring

Adverse events were assessed by CRC nurses and study physicians at each visit. Participants were asked to keep a detailed diary to record new symptoms, time of administration INI/placebo administration, concomitant medications, meals, and activities [10].

### Long-term INI Safety

The long-term INI safety, glucose metabolism and hypoglycemic episodes were measured at baseline, on-treatment, and post-treatment by fasting plasma glucose, HbA1c, fasting plasma insulin, vital signs, and weight at assessment visits during on-treatment and post-treatment. Long-term outcomes were metabolic parameters and hypoglycemic episodes.

#### Metabolic measurements:

Fasting capillary SMBG was measured weekly before breakfast during treatment period. Fasting plasma glucose, fasting capillary glucose, HbA1c, fasting plasma insulin, blood pressure, heart rate, and body weight were measured at baseline, on-treatment and post-treatment study visits.

Hypoglycemia monitoring: HG was monitored using weekly SMBG and fasting laboratory panels during study visits.

## Data Management and Statistical Analysis

MemAID database (Study TRAX© Macon, GA, USA; https://my.studytrax.com) is FDA and the Health Insurance Portability and Accountability Act (HIPAA) compliant web-based data management software. It was used for the data storage, management, coding, and verification. Data are presented as mean and SD for INI and placebo groups and also individually for each participants. Short-term safety outcomes (CGM, SMBG glucose and glycemic variability) were compared between baseline and intervention period using t-test (α = 0.05) within each participant. Long-term safety outcomes were collected at baseline, during on-treatment visits (V2–V8; average days from baseline visit 2: 0, 1, 55, 113, 165, respectively) and during post-treatment visits (V9 – V12; average days from baseline visit 2: 173, 227, 282, 333). Safety measures were characterized using descriptive statistics and were compared between two INI and three Placebo treated participants at baseline, on-treatment and post-treatment. Spatial power mixed-models were used, and each variable was modeled separately. The independent variables in the model included the two treatment groups, a time indicator variable (TIMEG) representing baseline, on-treatment and post-treatment period and an interaction term between TIMEG and treatment group. An average number of treatment days at each assessment visit was used as a continuous repeated variable and subjects were included as random effects. Restricted maximum likelihood estimation method and linear contrasts were used to obtain the estimated mean difference and 95% confidence interval between INI and Placebo for each outcome variable. Adverse events and hypoglycemic episodes are described.

Data were converted from Study TRAX© (Macon, GA, USA). The code and data analyses were generated using Statistical Analyses Software (SAS), Version 9.4 TS level; SAS System for Windows (X64_8PRO platform, Copyright © 2002–2012 SAS Institute Inc. (Cary, NC, USA) and JMP® Pro, Version 15 (SAS Institute Inc. Cary, NC, USA).

## Results

### Participant flow and Baseline Characteristics

**Figure 1:**
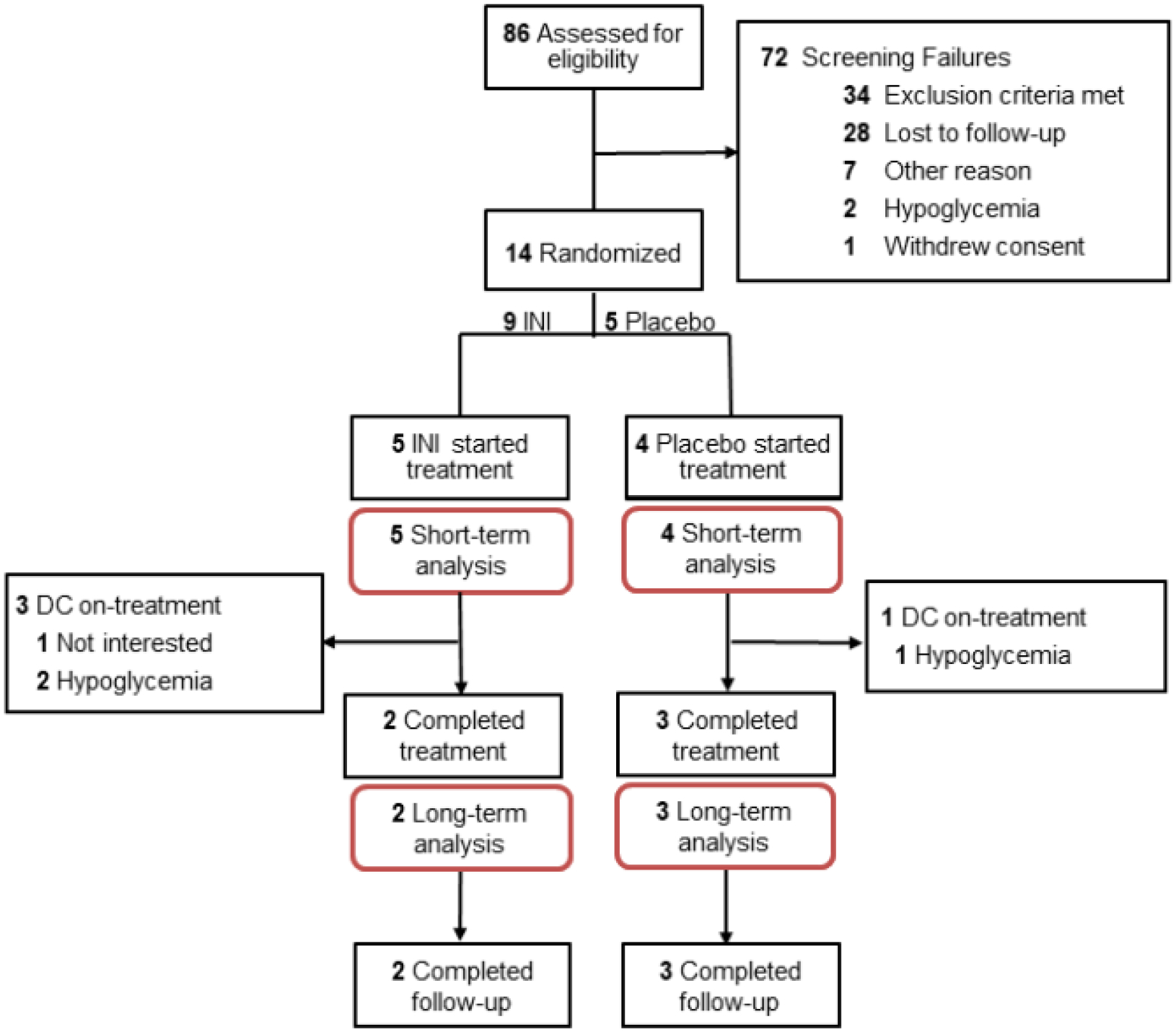
CONSORT diagram.

A total of 86 participants with T2DM on systemic insulin were assessed for eligibility, 61 unsuccessful phone screenings, of which 40 were due to lack of interest (26 not interested, 14 lost to follow-up). 25 participants signed informed consent. 11 were excluded before randomization, 10 of them met exclusion criteria such as HG, participation in another study or new onset medical condition, and one was no longer interested in participating. 14 were eligible, signed informed consent and were randomized. Five participants were discontinued before treatment. Four participants withdrew consent due to lack of interest in the study and one participated in another research study. Nine participants started treatment, five INI and four Placebo (INI 1–5; PL 3–6) and five participants, two INI and three Placebo (INI 1&2 and PL 3–5) completed treatment and post-treatment periods. (Figure 1). Demographic characteristics of nine subjects who were eligible, enrolled, and started INI or placebo are summarized in the Table 1. Individual baseline and demographic data for nine randomized participants are available in the Table S1 of Supplementary Appendix.

### Short-term INI Safety

#### Continuous Glucose Monitoring

**Figure 2(A-B):**
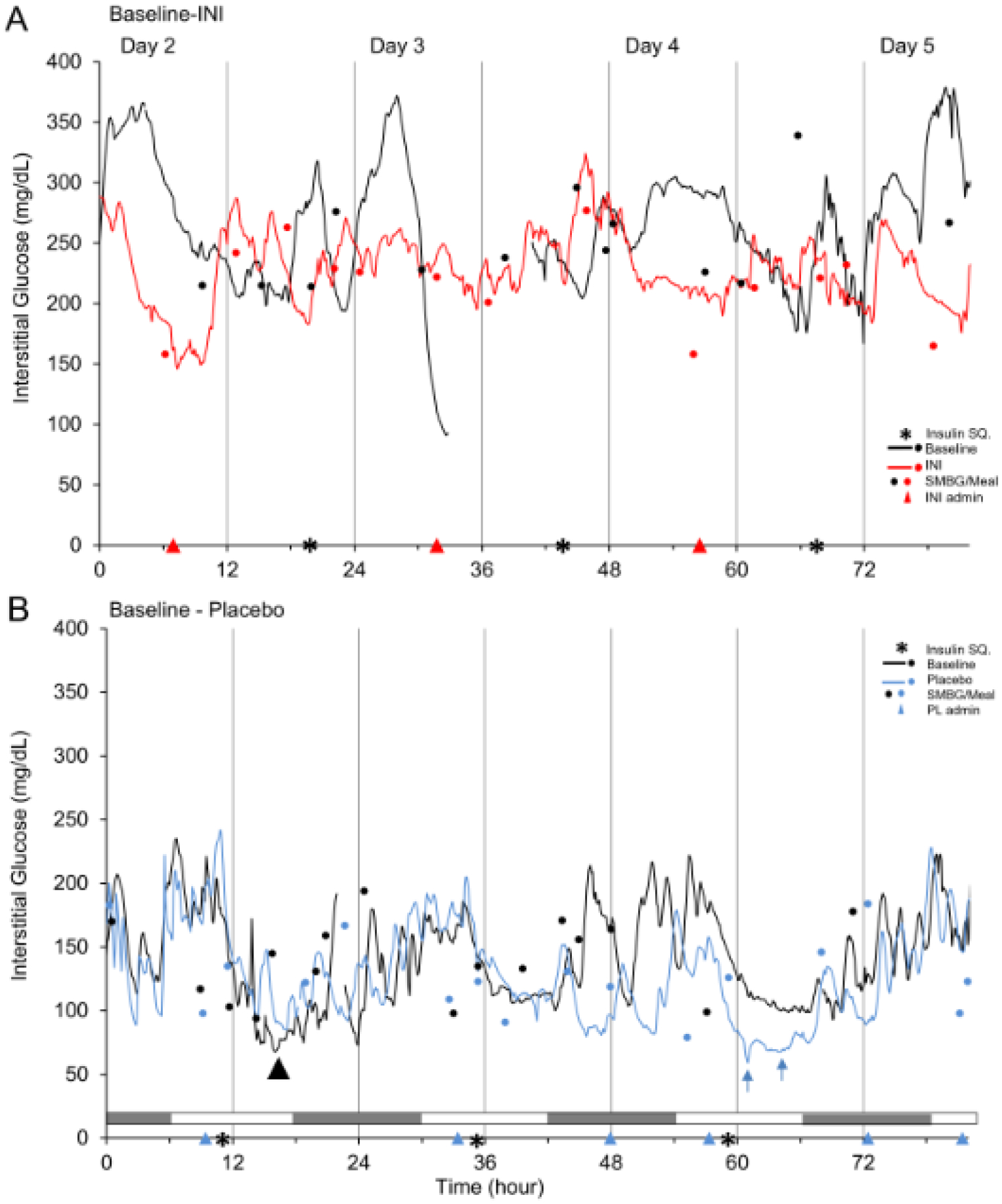
Interstitial glucose during CGM recordings for four days at baseline and after treatment initiation. **Figure 2A:** INI-treated T2DM participant on systemic insulin (INI-1) completed CGM monitoring for four days with SMBG at baseline and after INI treatment initiation. Baseline CGM: black line, SMBG before meals: black circle; INI-CGM: red line, SMBG before meals: red circle, INI administration: red triangle; subcutaneous insulin administration: black asterisk. CGM data displayed from Day 2 at midnight. There was a sensor malfunction on Day-3. **Figure 2B:** Placebo-treated T2DM participant on systemic insulin (PL-4), completed CGM monitoring for four days with SMBG at baseline and after placebo treatment initiation. Baseline CGM: black line, SMBG before meals: black circle; Placebo-CGM: blue line, SMBG before meals: blue circle, Placebo administration: blue triangle; subcutaneous insulin administration: black asterisk. There was one Level I hypoglycemic episode on Day 2 at baseline (black arrow) and two Level I hypoglycemic episodes on placebo treatment on Day 4 (blue arrows). CGM data displayed from Day 2 at midnight. Day-night (white and gray horizontal bar).

Nine randomized participants were included in the analyses, of which seven completed CGM monitoring and two completed SMBG monitoring for five days prior to and after first dose of INI or placebo administration. To characterize the immediate effects of INI administration, we analyzed glycemic profiles at baseline and after treatment initiation. Figure 2 shows details of CGM recording of one participant in each group (Figure 2A & 2B) for four days before and four days after INI or placebo initiation. INI was administered in the morning before breakfast. INI administration did not affect interstitial or capillary glucose or caused hypoglycemic episodes or interacted with systemic insulins. INI-treated participant (INI-1) has demonstrated a greater glycemic variability at baseline as compared to intervention recording (Figure 2A). INI administration did not induced HG. Placebo-treated participant (PL-4) experienced one hypoglycemic episode at baseline and two during placebo treatment, but glycemic variability was similar (Figure 2B). Hypoglycemic episodes followed administration of prescribed subcutaneous insulin.

Table 2 summarizes SMBG, CGM, and indicators of glucose variability for five days prior to and after INI or placebo treatment initiation. Three out of five subjects in the INI group (INI-1, INI-4, INI-5) presented lower interstitial glucose levels on INI-intervention on CGM as compared to baseline (p<0.0001), one of them (INI-4) also showed a significant decrease of SMBG levels. Two out of four subjects in Placebo group (PL-4, PL-6) also presented decline in capillary glucose on CGM from baseline to intervention (p<0.001), one of them (PL-6) also showed a difference in SMBG. Participants INI-2 and PL-5 did not complete CGM due to early termination of the safety sub-study. Intra-day GV measures did not show significant differences between baseline and intervention in either group. Two participants in the INI group (INI-3, INI-5) had a higher MAGE index and two participants (INI-1, INI-4) had a lower MAGE index during intervention as compared to baseline. All participants in placebo group (PL-3, PL-4, PL-6) had higher intraday GV, MAGE index and two had also higher CV during intervention period. Differences in CONGA index of intervention compared to baseline were −0.7 in INI group and −1.4 in the Placebo group. Inter-day GV measured by MODD did not show major differences between intervention periods (INI group= −0.2 vs. Placebo group= 0.3). The difference in coefficient of variance (CV) on INI-treatment was 3.2% and 12.2% on placebo-treatment as compared to baseline.

Data are mean ± SD. P values were obtained by unpaired t-test comparing baseline and intervention period for each participants. Number of high glucose excursions (# HGE) grouped and duration (only for CGM) are listed per participant. Mean amplitude of glycemic excursions (MAGE), continuous overlapping net glycemic action (CONGA), mean of daily differences (MODD), and coefficient of variation (CV). Self-monitored blood glucose (SMBG) represents capillary glucose by finger stick for calibration of CGM.

### Long-term INI Safety

#### Metabolic outcomes

**Figure 3:**
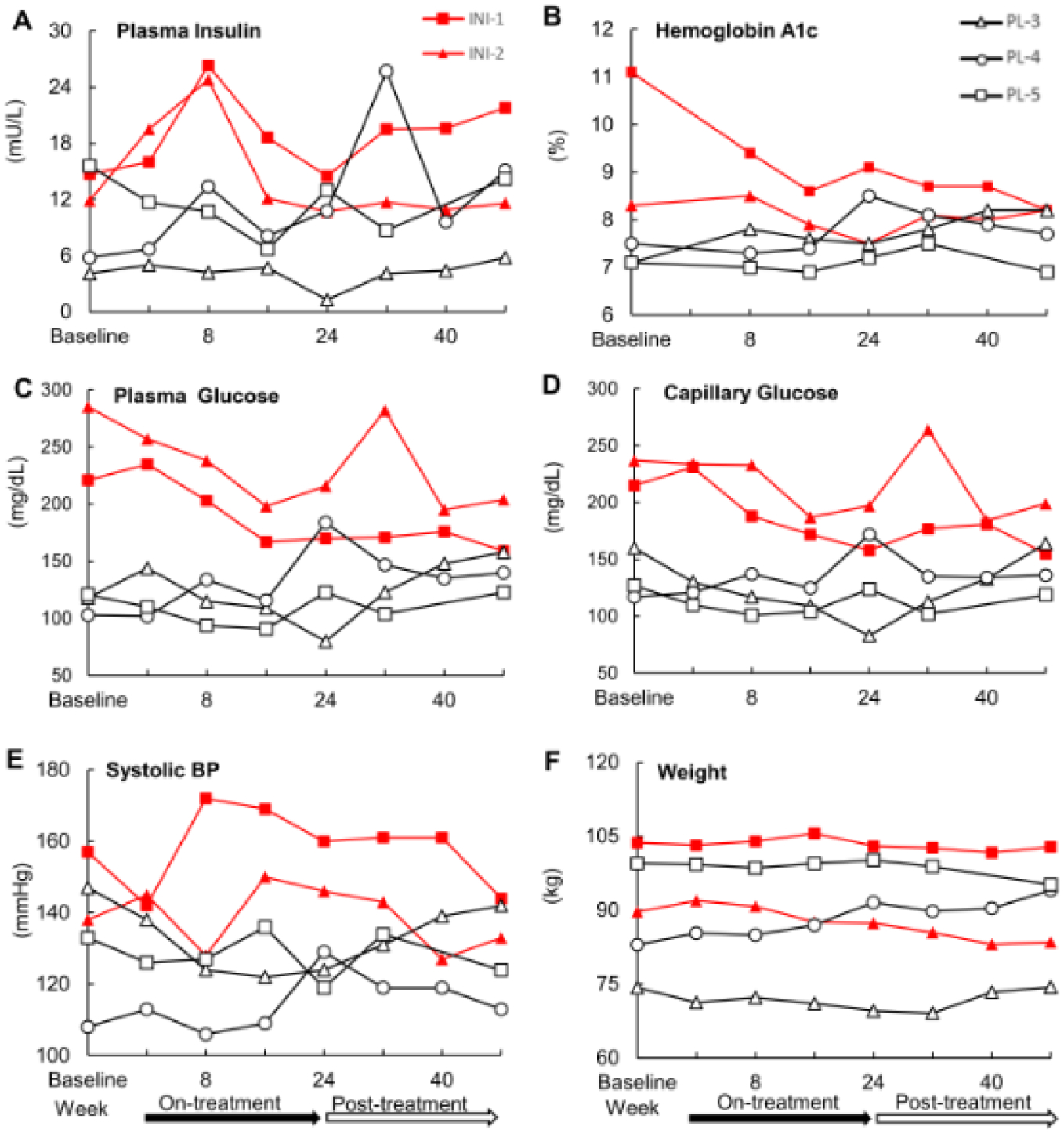
Long-term safety outcome measures. Safety measures over 24 week on-treatment and 24 weeks of post-treatment follow-up: hemoglobin A1c (A), fasting plasma glucose (B), fasting plasma insulin (C), capillary glucose (D), systolic blood pressure (BP; E) and weight (F) at baseline, on-treatment (week 2–24) and post-treatment (week 25–48) in two intranasal insulin treated (INI) (red lines) and three placebo-treated participants (black lines).

Five participants with T2DM on systemic insulin completed the study. Figure 3 displays the individual longitudinal profiles of fasting plasma insulin (A), HbA1c (B), fasting plasma glucose (C), fasting capillary glucose (D), systolic blood pressure (E), and body weight (F) at baseline, during treatment (week 1 to 24), and post-treatment (week 25 to 48) periods in two INI-treated participants (INI-1, INI-2) and three placebo-treated participants (PL-3,PL-4, PL-5). Table S3 presents long-term outcome variables for each participant. Both participants in the INI group showed tighter glycemic control (a decrease in HbA1c, fasting glucose and plasma insulin) during treatment period that was not observed in the Placebo group. Table S3 shows the differences and mixed model estimates between INI and Placebo participants at baseline, on-treatment and post-treatment. INI participants had higher baseline HbA1c (p=0.015), fasting glucose (p<0.001), but these values declined during on-treatment and post-treatment, and differences were no longer significant. The difference plasma glucose model estimate between INI and Placebo participants declined on-treatment by 44.89 mg/dL (36%) and by 62.71 mg/dL (50%). Plasma insulin was borderline higher at baseline but declined during on-treatment (p=0.002) and post-treatment (p=0.03). The difference plasma insulin model estimate between INI and Placebo participants declined on-treatment by 0.22 mIU/L (2.2%) and by 3.67 mIU/L (36.91%). Diabetes treatment regimen was adjusted in participant INI-1 post-treatment (week 32) and in participant INI-2 at the end of treatment period (week 22) together with a weight loss program. There were no notable changes in body weight, BMI, waist circumference, sitting heart rate, systolic or diastolic blood pressure in either group.

#### Hypoglycemia and other adverse events

All hypoglycemic episodes (Table S2), in both INI and placebo groups, herein reported were asymptomatic. INI-administration did not cause acute HG within two hours of CGM monitoring (Figure 2A), nor was associated with increased number of HG as compared to baseline and placebo treatment. INI was well tolerated and there were no treatment-related serious adverse events (AEs). Three participants (INI-3, INI-5, PL-5) were discontinued for HG after treatment initiation as per our criteria for discontinuation (i.e. participants who had more than one asymptomatic and/or symptomatic episode of Level-1 HG (glucose ≤ 70 mg/dL) on plasma glucose, SMBG or CGM during the entire study) and one withdrew consent (INI-4). All hypoglycemic episodes were observed on CGM, except for one Level-one HG recorded on SMBG (PL-5). No hypoglycemic episodes were observed during post-treatment period in either group. For INI and Placebo group, incidence of participants with any level of HG during treatment period was 40% and 75%, respectively. All AEs resolved without sequelae.

Figures 2A & 2B depict 4-day CGM trends before treatment initiation (baseline) as compared to and after treatment initiation (Placebo - INI) for one participant in each treatment group. In the INI group, there were no hypoglycemic episodes at baseline. During INI treatment, three participants (INI 3–5) experienced two level-one and four level-two hypoglycemic episodes on CGM. Participant INI-3 had one episode of level-1 HG and one episode of level-2 HG, which occurred from 5:00 to 6:00 AM and from 19:00 to 21:00 PM, respectively, and participant INI-5 had three episodes of level-2 HG around 4:00 to 9:00 AM before administration of INI, both participants were excluded per our pre-determined exclusion criteria. Hypoglycemic events did not occur within two hours after INI administration (Figure 2A & 2B). In the INI group, one participant (INI-4) presented one mild AE possibly related to the study protocol (skin irritation during CGM removal) and one participant (INI-1) reported one mild AE, unlikely related to the study (non-dysenteric diarrhea) during post-treatment period.

In the Placebo group, three participants (PL-3, PL-4, PL-5) experienced three level-one HG episodes at baseline. During placebo treatment, two participants (PL-4, PL-6) had two episodes, each of level-1 HG, and two participants (PL-3, PL-4) had one episode, each of level-2 HG. Participant PL-3 presented level-2 HG in the afternoon, and PL-4 presented HG episodes during sleeping time. Diabetes regimen for Participant PL-3 was Levemir 50 units at bedtime and Novolog sliding scale ranging from 6 to 16 IU with meals and Metformin. Participant PL-4 was on Lantus sliding scale ranging from 15 to 20 IU after morning SMBG and Canagliflozin 300 mg daily. One participant in Placebo group (PL-4) reported one mild AE (one episode of sinusitis) during post-treatment period, unlikely related as it occurred 18 weeks post-treatment.

## Discussion

The MemAID was a 24-week trial of INI treatment with 24-weeks of follow-up, and this Safety sub-study in subcutaneous insulin treated T2DM has demonstrated the long-term safety and feasibility of this emerging treatment of diabetes complications in the brain. MemAID trial showed that INI-treated control participants (i.e. without T2DM) demonstrated better executive functioning, verbal memory, and somewhat faster normal walking speed as compared to placebo. Participants with T2DM treated with INI had faster walking speed, increased cerebral blood flow on fMRI and lower plasma insulin and insulin resistance as compared T2DM on placebo [9]. INI therapy was safe in older diabetic participants treated with systemic insulins, it did not cause HG, nor disturbances in metabolic parameters and vital signs or weight gain. INI was not associated with moderate or serious adverse events. CGM demonstrated that INI had no acute effects on interstitial glucose within two hours of administration, and that there was no association between INI and HG. The incidence of HG was similar the INI and Placebo-treated participants.

This older T2DM population on subcutaneous insulin has different metabolic profiles, intensive glucose control therapy and higher number of systemic complications. Therefore, they are at greater risk for diabetes related cognitive and functional decline, which is further exaggerated by the presence of hypoglycemic episodes, HG unawareness and subsequently a higher risk of falls, and dementia [21]. Glycemic variability has been recognized an important risk factor for long-term complications of T2DM, brain atrophy, and cognitive decline [22]. Insulin, when administered intranasally using devices that deliver the drug in the upper nasal cavity, enters rapidly into the brain through perineural, vascular and lymphatic pathways, bypassing blood-brain barrier [23–25]. It binds to insulin receptors throughout the brain, including hypothalamus, hippocampus, and insular cortex modulating signaling in the functional networks [26–28] and increasing brain energy metabolism [29]. Furthermore, it may increase central homeostatic regulation and peripheral insulin sensitivity [30]. In our study, short-term analyses of CGM recordings before and after INI/placebo administration showed that INI was associated with lesser glycemic variability as compared to placebo, as indicated by changes of mean glucose levels with a similar trend on CONGA and MAGE. Long-term-analyses results also suggested a downward trend in HbA1c, fasting glucose and plasma insulin over six months period, which was also observed in the analysis of INI-treated T2DM in MemAID trial [9].

The main limitation of the present long-term study was recruitment and retention of older participants with T2DM, namely because of functional difficulties and co-morbidities that interfere with adherence to study procedures and visits. Despite of initial enthusiasm of 86 participants screened over the phone, only 14 participants were randomized and 5 completed the study. The rate of screening failures was due to lack of interest (44% of screening failures), complex medical conditions (50%) and HG (5.8%) as per our tight criteria triggering discontinuation. Complexity of the study procedures may have contributed to the lack of interest in participation. We had strict safety criteria and excluded participants with more than one HG during entire study on CGM, SMBG or plasma. Five participants were excluded for HGs that were unrelated to INI and study protocol (two during screening, three after randomization before initiation of INI/placebo). In further studies, it would be useful to separate subcutaneous insulin administration from INI administration in order to clearly separate HG related to systemic insulin treatment from those possibly related to INI. INI was administered in the morning before breakfast and it can be detected in cerebrospinal fluid for about two hours [31], and therefore hypoglycemic episodes occurring at different times are unlikely related to INI. Small sample size of this sub-study make difficult to interpret potential INI effects in the context of adjustments of anti-diabetic regimens and effects of more frequent glucose monitoring during study participation on outcomes of interest.

Older T2DM participants on systemic insulins are among the most vulnerable population with multiple comorbidities, and therefore are at a high risk of diabetic complications in the brain, including stroke and dementia [1]. The potential effects of INI on glycemic variability are yet to be explored in further studies to determine whether a centrally mediated reduction of glycemic variance can bring benefits to long-term care of older diabetic patients, and potentially reduce the number of hypoglycemic episodes. The MemAID is the longest trial of INI treatment in subcutaneous insulin treated T2DM to date and demonstrated that INI therapy is safe in this elderly patient population. This pilot sub-study paves the way for future research needed to determine whether INI could be potentially beneficial by reducing glycemic variability and improving peripheral insulin sensitivity and thus potentially lessen diabetes burden in this population, as well as improving mobility and cognitive outcomes in this vulnerable population.

## Supplementary Material

1

## Figures and Tables

**Table 1:** Baseline and demographic characteristics of randomized participants.Data are mean ± SD, or percentage. (BMI) Body mass index; (HbA1c) Hemoglobin A1c.

	T2DM on Systemic Insulin	INI	Placebo
Number of participants-baseline	9	5	4
Age, years Range	64.8 ± 7.3	61.4 ± 8.6	69.0 ± 1.4
	52–75	52 – 75	68 – 71
Women, n (%)	3 (33)	2 (40)	1 (25)
Diabetes duration, years range	12.1 ± 8.3	9.6 ± 10.2	15.3 ± 4.6
	5–27	5 – 27	9–20
White, n (%)	7 (78)	3 (60)	4 (100)
Black, n (%)	1 (11)	1 (20)	0 (0)
Asian, n (%)	0 (0)	0 (0)	0 (0)
Other, n (%)	1 (11)	1 (20)	0 (0)
Ethnicity - Hispanic, n (%)	1 (11)	1 (20)	0 (0)
Mini-Mental State Exam (scale 0–30)	28.1 ± 1.8	28.4 ± 1.8	27.8 ± 2.1
Charlson Comorbidity Index (scale 0–24)	4.0 ± 1.3	4.4 ± 1.7	3.5 ± 0.6
BMI, kg/m2	32.8 ± 6.5	34.1 ± 8.4	31.3 ± 3.5
Waist circumference, cm	115.1 ± 12.5	118.1 ± 15.3	111.4 ± 8.4
HbA1c, % HbA1c, mmol/mol	8.5 ± 1.5	9.4 ± 1.5	7.4 ± 0.4
	69.4 ± 16.2	79.0 ± 15.9	57.4 ± 4.2
Fasting serum glucose, mg/dL	146.8 ± 60.6	163.2 ± 78.5	126.3 ± 23.7
Capillary glucose, mg/dL	142.6 ± 48.3	159.5 ± 63.4	125.8 ± 25.9
Plasma insulin, mg/dL	31.4 ± 63.0	50.7 ± 83.0	7.4 ± 3.0
Fructosamine, umol/L	294.7 ± 27.5	304.2 ± 23.6	282.8 ± 30.5
C-reactive protein, mg/L	3.5 ± 2.1	3.5 ± 3.7	3.5 ± 1.4
Total cholesterol, mg/dL	157.6 ± 23.0	163.0 ± 30.7	150.8 ± 6.7
Microalbumin urine, mg/dL	25.6 ± 39.2	37.0 ± 51.6	11.3 ± 7.8
Microalbumin-to-creatinine ratio, mg/dL	18.8 ± 23.0	26.2 ± 29.9	9.5 ± 4.0
Hypertension diagnosis, n (%)	8 (89)	5 (100)	3 (75)
Hypertension duration, years	17.4 ± 6.5	14.6 ± 6.4	22.0 ± 3.6
Heart rate, bpm	70.8 ± 10.3	69.8 ± 11.6	72.0 ± 10.0
Systolic blood pressure, mmHg	135.4 ± 11.3	142.2 ± 6.8	127.0 ± 10.6
Diastolic blood pressure, mmHg	70.0 ± 15.4	67.2 ± 19.7	73.5 ± 9.1
Use of sq. insulin, n (%)	9 (100)	5 (100)	4 (100)
Use of oral antidiabetic drugs, n (%)	7 (78)	3 (60)	4 (100)
Use of injectable antidiabetic drugs, n (%)	2 (22)	0 (0)	2 (50)
Use of antihypertensive drugs, n (%)	8 (89)	5 (100)	3 (75)
Use of lipid lowering drugs, n (%)	6 (67)	3 (60)	3 (75)
Use of antidepressants, n (%)	2 (22)	0 (0)	2 (50)

**Table 2:** Short-term safety outcomes.

Intranasal Insulin																		
	Baseline									Intervention								
	INI-1		INI-2		INI-3		INI-4		INI-5	INI-1		INI-2		INI-3		INI-4		INI-5
**SMBG glucose, mg/dL**																		
# Recordings	14		9		15		20		15	15		21		19		21		20
Mean ± SD	250.5 ± 36.8		195.6 ± 65.8		136.5 ± 30.4		190.6 ± 59.7		271.6 ± 65.8	223.1±39.8		227.1±68.3		138.5 ± 37.3		242.1±75.7		220.9± 9.3
p-value										0.064		0.251		0.865		0.02		0.061
**CGM – Interstitial, mg/dL**																		
# Recordings	924		842		1045		1425		1011	1052		N/A		1430		1338		1436
Mean ± SD	265.4 ± 53.1		186.9 ± 61.2		150.8 ± 30.4		186.2 ± 51.6		269.7 ± 67.2	231.4±31.8		N/A		150.8±44.0		223.4±67.9		209.8±84.4
p-value										<0.0001				0.984		<0.0001		<0.0001
# HGE episodes	2		N/A		5		16		12	2		N/A		15		8		18
HGE duration	74:25:00		N/A		13:40		88:25:00		74:45:00	83:40:00		N/A		29:40:00		93:50:00		89:35:00
**Glucose Variability**																		
MAGE	6.7		9.4		4.1		5.9		3.2	3.8		N/A		5.1		5.1		6.5
CONGA	13.7		9.3		7.7		9.1		13.7	12		N/A		7.4		11.5		10.5
MODD	2.9		3.1		1.4		3.3		4.3	2		N/A		2.1		4.2		4.3
CV%	20		32.7		20.2		27.7		24.9	13.7		N/A		29.2		30.4		40.2
Placebo																		
		Baseline									Intervention							
		PL-3		PL-4		PL-5		PL-6			PL-3		PL-4		PL-5		PL-6	
**SMBG glucose, mg/dL**																		
# Recordings		24		25		24		22			7		18		12		13	
Mean ± SD		167.5 ± 66.6		149.5 ± 48.7		137.1 ± 33.5		207.9 ± 61.9			203 ± 69.7		131.5 ± 40.0		152.7 ± 40.4		151.7 ± 66.1	
p-value											0.261		0.191		0.265		0.02	
**CGM- Interstitial, mg/dL**																		
# Recordings		1427		1403		N/A		1694			374		1132		N/A		1020	
Mean ± SD		159.1 ± 41.8		150.6 ± 43.0		N/A		220.4 ± 63.4			158.1 ± 81.7		130.5 ± 35.5		N/A		146.3± 60.6	
p-value											0.809		<0.0001				<0.0001	
# HGE episodes		11		21		N/A		10			4		12		N/A		7	
HGE duration		69:55:00		55:45:00		N/A		123:15:00			14:10		25:05:00		N/A		33:15:00	
**Glucose Variability**																		
MAGE		4.6		0.1		N/A		7.8			6.5		3.9		N/A		8.1	
CONGA		8		7.4		N/A		10.9			8.1		6.4		N/A		7.4	
MODD		2.5		1.9		N/A		3.7			N/A		1.9		N/A		3.1	
CV%		26.3		28.6		N/A		28.8			51.7		27.2		N/A		41.4	

## References

[1] Xu WL, Qiu CX, Wahlin A, Winblad B, Fratiglioni L (2004) Diabetes mellitus and risk of dementia in the Kungsholmen project: A 6-year follow-up study. Neurology 63(7): 1181–1186. 15477535 10.1212/01.wnl.0000140291.86406.d1

[2] Novak V, Milberg W, Hao Y, Munshi Medha, Novak Peter, (2014) Enhancement of vasoreactivity and cognition by intranasal insulin in type 2 diabetes. Diabetes Care 37(3): 751–759. 24101698 10.2337/dc13-1672 PMC3931384

[3] Zhang H, Hao Y, Manor B, Novak Peter, Milberg William, (2015) Intranasal insulin enhanced resting-state functional connectivity of hippocampal regions in type 2 diabetes. Diabetes 64(3): 1025–1034. 25249577 10.2337/db14-1000 PMC4338591

[4] Benedict C, Hallschmid M, Hatke A, Schultes Bernd, Fehm Horst L, (2004) Intranasal insulin improves memory in humans. Psycho neuroendocrinology 29(10): 1326–1334. 10.1016/j.psyneuen.2004.04.003 15288712

[5] Craft S, Claxton A, Baker LD, Hanson Angela J, Cholerton Brenna, (2017) Effects of Regular and Long-Acting Insulin on Cognition and Alzheimer’s Disease Biomarkers: A Pilot Clinical Trial. Journal of Alzheimer’s Disease 57(4): 1325–1334. 10.3233/JAD-161256 PMC5409050 28372335

[6] Kellar D, Craft S (2020) Brain insulin resistance in Alzheimer’s disease and related disorders: Mechanisms and therapeutic approaches. The Lancet Neurology 19(9): 758–766. 32730766 10.1016/S1474-4422(20)30231-3 PMC9661919

[7] Benedict C, Dodt C, Hallschmid M, Lepiorz Marc, Fehm Horst L, (2005) Immediate but not long-term intranasal administration of insulin raises blood pressure in human beings. Metabolism: Clinical and Experimental 54(10): 1356–1361. 16154436 10.1016/j.metabol.2005.04.026

[8] Schmid V, Kullmann S, Gfrorer W, Hund Verena, Hallschmid Manfred, (2018) Safety of intranasal human insulin: A review. Diabetes, Obesity & Metabolism 20(7): 1563–1577. 10.1111/dom.13279 29508509

[9] Novak V, Mantzoros CS, Novak P, McGlinchey Regina, Dai Weiying, (2022) MemAID: Memory advancement with intranasal insulin vs. placebo in type 2 diabetes and control participants: A randomized clinical trial. J Neurol 1–9. 35482079 10.1007/s00415-022-11119-6 PMC9046533

[10] Galindo-Mendez B, Trevino JA, McGlinchey R, Fortier C, Lioutas V, (2020) Memory advancement by intranasal insulin in type 2 diabetes (MemAID) randomized controlled clinical trial: Design, methods and rationale. Contemporary Clinical Trials 89: 105934. 31923471 10.1016/j.cct.2020.105934 PMC7242142

[11] Seaquist ER, Anderson J, Childs B, Cryer Philip, Jack Samuel Dagogo, (2013) Hypoglycemia and Diabetes: A Report of a Workgroup of the American Diabetes Association and The Endocrine Society. Diabetes Care 36(5): 1384–1395. 23589542 10.2337/dc12-2480 PMC3631867

[12] Danne T, Nimri R, Battelino T, Bergenstal Richard M, Close Kelly L, (2017) International Consensus on Use of Continuous Glucose Monitoring. Diabetes Care 40(12): 1631–1640. 29162583 10.2337/dc17-1600 PMC6467165

[13] Vaddiraju S, Burgess DJ, Tomazos I, Jain FC, Papadimitrakopoulos F (2010) Technologies for Continuous Glucose Monitoring: Current Problems and Future Promises. J Diabetes Sci Technol 4(6): 1540–1562. 21129353 10.1177/193229681000400632 PMC3005068

[14] Maiorino MI, Signoriello S, Maio A, Chiodini Paolo, Bellastella Giuseppe, (2020) Effects of Continuous Glucose Monitoring on Metrics of Glycemic Control in Diabetes: A Systematic Review with Meta-analysis of Randomized Controlled Trials. Diabetes Care 43(5): 1146–1156. 32312858 10.2337/dc19-1459

[15] Allen NA, Fain JA, Braun B, Chipkin SR (2008) Continuous glucose monitoring counseling improves physical activity behaviors of individuals with type 2 diabetes: A randomized clinical trial. Diabetes Res Clin Pract 80(3): 371–379. 18304674 10.1016/j.diabres.2008.01.006 PMC2430041

[16] 7. Diabetes Technology: Standards of Medical Care in Diabetes-2021, Diabetes Care, American Diabetes Association. 10.2337/dc21-S007 33298418

[17] Weber C, Schnell O (2009) The assessment of glycemic variability and its impact on diabetes-related complications: an overview. Diabetes Technol Ther 11(10): 623–633. 19821754 10.1089/dia.2009.0043

[18] Dadlani V, Kudva YC (2017) Assessment of Interday Glucose Variability in Type 2 Diabetes. Diabetes Technol Ther 19(8): 443–445. 28817341 10.1089/dia.2017.0255 PMC5568177

[19] Hill NR, Oliver NS, Choudhary P, Levy JC, Hindmarsh P, (2011) Normal reference range for mean tissue glucose and glycemic variability derived from continuous glucose monitoring for subjects without diabetes in different ethnic groups. Diabetes Technol Ther 13(9): 921–928. 21714681 10.1089/dia.2010.0247 PMC3160264

[20] American Diabetes Association (2019) 6 Glycemic Targets: Standards of Medical Care in Diabetes-2019. Diabetes Care 42(Supplement 1): 61–70. 10.2337/dc19-S006 30559232

[21] Shafiee G, Mohajeri Tehrani M, Pajouhi M, Larijani B (2012) The importance of hypoglycemia in diabetic patients. J Diabetes Metab Disord 11(1): 17. 23497433 10.1186/2251-6581-11-17 PMC3598174

[22] Cui X, Abduljalil A, Manor BD, Peng CK, Novak V (2014) Multi-scale glycemic variability: a link to gray matter atrophy and cognitive decline in type 2 diabetes. PLoS One 9(1): 86284. 10.1371/journal.pone.0086284 PMC3901681 24475100

[23] Thorne RG, Pronk GJ, Padmanabhan V, Frey WH (2004) Delivery of insulin-like growth factor-I to the rat brain and spinal cord along olfactory and trigeminal pathways following intranasal administration. Neuroscience 127(2): 481–496. 15262337 10.1016/j.neuroscience.2004.05.029

[24] Lochhead JJ, Kellohen KL, Ronaldson PT, Davis TP (2019) Distribution of insulin in trigeminal nerve and brain after intranasal administration. Scientific Reports 9(1): 2621. 30796294 10.1038/s41598-019-39191-5 PMC6385374

[25] Lochhead J, Thorne RG (2011) Intranasal Delivery of Biologics to the Central Nervous System. Advanced drug delivery reviews 64(7): 614–628. 22119441 10.1016/j.addr.2011.11.002

[26] Kullmann S (2013) Intranasal insulin modulates intrinsic reward and prefrontal circuitry of the human brain in lean women. Neuroendocrinology 97(2): 176–182. 22922661 10.1159/000341406

[27] Akintola AA, van Heemst D (2015) Insulin, Aging, and the Brain: Mechanisms and Implications. Front Endocrinol (Lausanne) 6: 13. 25705204 10.3389/fendo.2015.00013 PMC4319489

[28] Edwin Thanarajah S, Iglesias S, Kuzmanovic B, Rigoux Lionel, Stephan Klaas E, (2019) Modulation of midbrain neurocircuitry by intranasal insulin. Neuroimage 194: 120–127. 30914385 10.1016/j.neuroimage.2019.03.050

[29] Jauch Chara K, Friedrich A, Rezmer M, Melchert Uwe H, Scholand-Engler Harald G, (2012) Intranasal Insulin Suppresses Food Intake via Enhancement of Brain Energy Levels in Humans. Diabetes 61(9): 2261–2268. 22586589 10.2337/db12-0025 PMC3425399

[30] Heni M, Kullmann S, Ketterer C, Guthoff M, Linder K, (2012) Nasal insulin changes peripheral insulin sensitivity simultaneously with altered activity in homeostatic and reward-related human brain regions. Diabetologia 55(6): 1773–1782. 22434537 10.1007/s00125-012-2528-y

[31] Born J, Lange T, Kern W, McGregor GP, Bickel U, (2002) Sniffing neuropeptides: A transnasal approach to the human brain. NatNeurosci 5(6): 514–516. 10.1038/nn849 11992114

