# Supplementary material for: Safety of Intranasal Insulin in Type 2 Diabetes on Systemic Insulin: A Double-Blinded Placebo-Controlled Sub-Study of Memaid Trial
Source: Arch Diabetes Obes. Author manuscript; Available in PMC 2022 Jul 27. (PMC9328174)
Supplement: 1 [file NIHMS1821832-supplement-1.pdf]

Table S1: Baseline demographic and metabolic characteristics by study participant.

Parameter	INI (n=5)					Placebo (n=4)			
	INI-1	INI-2	INI-3	INI-4	INI-5	PL3	PL4	PL5	PL6
Age, years	52	63	75	60	57	68	68	69	71
Gender	Male	Male	Female	Female	Male	Male	Female	Male	Male
Race	White	Black	White	White	Other	White	White	White	White
Hispanic or Latino	No	No	No	Yes	No	No	No	No	No
Diabetes duration, years	2	27	5	10	4	9	20	17	15
Mini-Mental State Exam (scale 0-30)	30	26	30	27	29	29	26	30	26
Charlson Comorbidity Index (scale 0-24)	5	4	7	3	3	3	4	3	4
Body Mass Index, kg/m2	30.7	29.9	24.3	40.8	44.7	26.9	35.5	31.6	31.1
Waist circumference, cm	113.4	111.9	100.3	123.6	141.1	98.9	114.7	117.4	114.6
HbA1c ,%									
HbA1c, mmol/mol	11.1	8.3	7.5	9.9	10.1	7.1	7.5	7.1	7.9

Citation: L Aponte Becerra, B Galindo Mendez, F Khan, V Lioutas, V Novak*. Safety of Intranasal Insulin in Type 2 Diabetes on Systemic Insulin: A Double-Blinded Placebo-Controlled Sub-Study of Memaid Trial. Archives of Diabetes & Obesity 4(2)- 2022. ADO.MS.ID.000182.
DOI: 10.32474/ADO.2022.04.000182

	97.8	67.2	58.5	84.7	86.9	54.1	58.5	54.1	62.8
Fasting serum glucose, mg/dL	235	257	85	139	100	144	102	110	149
Capillary glucose, mg/dL	158	219	72	N/A	189	163	123	112	105
Plasma insulin, mg/dL	16.0	19.5	2.7	16.5	198.7	5.0	6.7	11.7	6.0
Fructosamine, umol/L	335	320	281	303	282	316	261	253	301
C-reactive protein, mg/L	6.1	0.8	5.3	15.0	16.9	2.2	3.5	4.9	0.8
Total cholesterol, mg/dL	137	177	125	198	178	141	155	155	152
Microalbumin urine, ug/mL	126.7	11.0	3.0	34.8	9.5	16.0	6.6	3.0	19.6
Microalbumin/ creatinine ratio, mg/g creat	77.3	17.6	3.0	25.4	8.3	10.1	6.9	3.0	14.9
Hypertension diagnosis	Yes	Yes	Yes	Yes	Yes	Yes	Yes	Yes	No
Hypertension duration, years	6	16	16	16	24	16	19	14	N/A
Heart rate, bpm	67	80	51	78	73	73	69	85	61
Systolic blood pressure, mmHg	142	145	131	149	144	138	113	126	131
Diastolic blood pressure, mmHg	76	72	45	50	93	79	60	79	76
Use of sq. insulin	Yes	Yes	Yes	Yes	Yes	Yes	Yes	Yes	Yes
Use of oral antidiabetic drugs	Yes	Yes	Yes	No	No	Yes	Yes	Yes	Yes
Use of injectable antidiabetic drugs	No	No	No	No	No	No	Yes	Yes	No
Use of antihypertensive drugs	Yes	Yes	Yes	Yes	Yes	Yes	Yes	Yes	No
Use of lipid lowering drugs	Yes	Yes	Yes	No	No	Yes	Yes	Yes	No
Use of antidepressants	No	No	No	No	No	No	Yes	Yes	No

Table S3: Primary long-term safety outcomes.

INI: Intranasal Insulin; DM: Diabetes Mellitus.

Comparisons of primary outcomes between two INI-treated and three Placebo-treated participants at baseline, on-treatment and post-treatment using spatial power mixed models. Estimates, confidence intervals (CI) and p values are the differences between DM-INI and DM-Placebo at each period.

Variable	Baseline		On-treatment		Post-treatment	
	Estimate (CI)	p	Estimate (CI)	p	Estimate (CI)	p
INI vs. Placebo						
Hemoglobin A1c, %	1.97 (0.53-3.41)	0.015	0.94 (-0.63-2.51)	0.165	0.98 (-0.60-2.56)	0.152
Fasting serum glucose, mg/dL	127.33 (74.14-180.53)	<0.0001	82.44 (51.73-113.16)	<0.0001	64.62 (35.25-93.98)	<0.0001
Capillary glucose, mg/dL	112.17 (67.54-156.79)	<0.0001	70.39 (40.96-99.82)	<0.0001	62.25 (36.62-87.89)	<0.0001
Fructosamine, umol/L	42.00 (-7.20-91.19)	0.091	16.50 (-19.79-52.78)	0.331	32.54 (-3.46-68.54)	0.071
Insulin, mIU/L	9.95 (-0.42-20.32)	0.059	9.73 (3.74-15.72)	0.002	6.28 (0.55-12.00)	0.033
Systolic blood pressure, mmHg	19.37 (-2.49-41.22)	0.080	34.01 (16.66-51.37)	0.002	13.61 (-3.67-30.89)	0.108
Diastolic blood pressure, mmHg	2.09 (-14.47-18.64)	0.791	7.81 (-7.79-23.42)	0.264	9.89 (-5.77-22.55)	0.170
Weight, kg	9.38 (-25.56-44.31)	0.461	9.17 (-25.81-44.15)	0.470	8.15 (-26.83-43.13)	0.517

Table S2: Long-term safety outcomes during on-treatment and post-treatment periods by study participant.

INI: Intranasal Insulin PL: Placebo

Outcome	INI (n=2)				Placebo (n=3)					
	On-treatment		Post-treatment		On-treatment			Post-treatment		
	INI-1	INI-2	INI-1	INI-2	PL-3	PL-4	PL-5	PL-3	PL-4	PL-5
Fasting serum glucose, mg/dL	180.0 ± 20.0	217.3 ± 20.0	176.8 ± 17.7	227.0 ± 47.8	101.3 ± 18.7	144.7 ± 35.2	102.7 ± 17.7	138.5 ± 17.3	140.7 ± 6.0	113.5 ± 13.4
Capillary glucose, mg/dL	212.2 ± 42.5	187.2 ± 66.7	175.5 ± 14.5	205.8 ± 40.0	152.8 ± 31.8	114.8 ± 27.6	120.4 ± 21.6	133.0 ± 22.2	135.0 ± 1.0	115.3 ± 11.9
Hemoglobin A1c ,%	9.0 ± 0.4	8.0 ± 0.5	8.7 ± 0.4	8.1 ± 0.1	7.6 ± 0.2	7.7 ± 0.7	7.0 ± 0.2	8.0 ± 0.3	7.9 ± 0.2	7.2 ± 0.4
Plasma insulin, mg/dL	19.8 ± 6.0	15.9 ± 7.8	20.7 ± 1.3	11.4 ± 0.4	3.4 ± 1.8	10.8 ± 2.7	10.1 ± 3.2	5.1 ± 1.0	16.8 ± 8.2	11.5 ± 3.9
Fructosamine, umol/L	310.3 ± 26.5	300.7 ± 11.4	301.3 ± 16.8	311.3 ± 18.2	290.3 ± 28.4	279.7 ± 45.7	287.3 ± 6.5	305.5 ± 11.0	257.0 ± 2.6	268.5 ± 33.2
BMI, kg/m2	31.1 ± 0.3	28.8 ± 0.6	30.7 ± 0.2	27.3 ± 0.4	26.8 ± 0.5	36.5 ± 1.4	31.7 ± 0.3	27.1 ± 1.0	38.1 ± 1.0	30.9 ± 0.8
Waist circumference, cm	114.8 ± 1.5	108.7 ± 2.4	112.0 ± 0.4	104.3 ± 3.7	98.5 ± 1.9	117.0 ± 2.5	117.0 ± 0.9	100.0 ± 1.5	118.9 ± 3.7	117.1 ± 0.1
Heart rate, bpm	75.7 ± 4.7	87.7 ± 5.9	72.3 ± 4.1	85.7 ± 13.0	74.0 ± 8.7	82.0 ± 5.2	85.7 ± 7.4	76.5 ± 2.5	81.3 ± 10.7	87.5 ± 4.9
Systolic blood pressure, mmHg	167.0 ± 6.2	141.3 ± 11.7	152.8 ± 9.5	134.3 ± 8.1	123.3 ± 1.2	114.7 ± 12.5	127.3 ± 8.5	136.3 ± 5.1	117.0 ± 3.5	129.0 ± 7.1
Diastolic blood pressure, mmHg	90.3 ± 3.2	79.3 ± 5.5	89.8 ± 6.7	81.3 ± 7.6	82.0 ± 2.0	64.7 ± 9.0	79.3 ± 1.5	80.5 ± 6.2	64.3 ± 1.5	76.0 ± 1.4
